# Parents’ planning, children’s agency and heritage language education: Re-storying the language experiences of three Chinese immigrant families in Australia

**DOI:** 10.3389/fpsyg.2022.1083813

**Published:** 2023-01-06

**Authors:** Chunxuan Shen, Wenying Jiang

**Affiliations:** ^1^Zhejiang Gongshang University Hangzhou College of Commerce, Hangzhou, Zhejiang, China; ^2^School of Languages and Cultures, The University of Queensland, Brisbane, QLD, Australia

**Keywords:** family language policy, heritage language, parent agency, child agency, Chinese immigrant families

## Abstract

This study delves into the heritage language experiences of Australian-born Chinese immigrant children under the framework of family language policy. Storytelling as a narrative inquiry method is used to reveal the lived experiences of the protagonists in relation to heritage language and culture. The three family stories involved for case studies reveal different levels of parent agency in Chinese immigrant families regarding their children’s home language use and heritage language education. It is noted that the level of child agency corresponds with the level of their parent agency. Where parents strongly advocate and practice heritage language maintenance, stronger agency is observed in their children to continue the use and learning of their heritage language. In addition, maintaining harmony while parents are implementing family language policies and providing children with formal instruction in heritage language are conducive to heritage language development, particularly in terms of its literacy.

## 1. Introduction

Drawing on three family stories, this study presents the findings of an ethnographic study on family language policy (FLP) in Chinese immigrant families in Brisbane, Australia. The paramount importance of family to the transmission of heritage language (HL) and culture has been acknowledged by a large number of researchers in recent decades (Tannenbaum and Howie, 2002; King et al., 2008; Fogle and King, 2013; Revis, 2019; Curdt-Christiansen and Huang, 2020; Wilson, 2020, etc.). Australia is a multilingual and multicultural country with “a strong and sustained history of immigration” (Collins, 2013, p. 134), which offers extensive opportunities for FLP research. Among all languages other than English (LOTE^1^) spoken in Australian households, Mandarin ranks first in the past Australian Censuses (Australian Bureau of Statistics, 2017). An in-depth investigation of how Mandarin is maintained in Chinese immigrant families may provide insights into the conundrum of reversing language shift for many other Australian community languages. Tannenbaum (2003, p. 374) advocates that the second-generation immigrants who were born and raised in Australia are the “transition generation” that hold the key to whether their HLs will be maintained or lost. This study is devoted to revealing a nuanced picture of FLPs in the three families and focalizing the critical role of parents’ planning and child agency in the enactment of FLPs. Agency, i.e., an individual’s “socioculturally mediated capacity to act” (Ahearn, 2001, p. 112), has received increasing scholarly attention in FLP studies. Parental agency, according to King et al. (2008), includes parents’ ideology, practice and management strategies in relation to HL, which, to a large extent, impacts children’s HL use and learning outcomes. Concomitantly, a growing body of research has recently highlighted the role of child agency in implementing, negotiating and adjusting FLPs (e.g., Fogle and King, 2013; Curdt-Christiansen and Huang, 2020; Smith-Christmas, 2020, 2022). Fogle and King (2013) argued that children could act as powerful agents in FLPs by making metalinguistic comments about language rules, using strategies to negotiate parental practices, or influencing parental responses to their developing bilingual/multilingual competence. Their research, therefore, makes an urgent call for more scholarly attention to be placed on the role of children in FLP studies.

## 2. Definition of heritage language learners

Heritage language learning has long been recognized as a topic of significance in bilingual research. The term “heritage language” is often employed to denote a socio-politically minority language acquired by children in the home environment either as a first language since birth or developed simultaneously with a dominant language of a larger society (Montrul, 2018). It is also called “home language,” “family language,” “minority language,” “maternal heritage language,” “mother tongue” or “community language” by different researchers (Clyne and Kipp, 2006; Montrul, 2018; Sun, 2019; Curdt-Christiansen and Huang, 2020; Smith-Christmas, 2020; Sun et al., 2022). These terms denote its “particular family relevance” (Fishman, 2001, p. 169), “heritage connection to the language” (Cummins, 2005, p. 586), parental influence (Sun, 2019) and its weaker status as opposed to the majority language in the society (Clyne and Kipp, 2006). Therefore, the acquisition of HL heavily relies on home language environment, parents’ HL proficiency and use, as well as community and educational support (Sun et al., 2020, 2022).

Due to the quantity and quality of HL input and a variety of internal and external factors (Sun et al., 2020), HL learners’ proficiency in HL may vary greatly from a very basic level of understanding daily home communication to a full and literate proficiency in both HL and the dominant language of the society (Gibbons and Ramirez, 2004; Hayakawa et al., 2022). Given the wide range of HL proficiency, some scholars also defined HL learners from the angle of agency instead of their competency or proficiency in HL. For instance, in Hornberger and Wang’s (2008) definition, HL learners are “individuals with familial or ancestral ties to a language other than English who exert their agency in determining if they are heritage language learners of that language” (p. 6). Their definition places more emphasis on the learners’ initiatives, self-positioning and self-negotiation in identifying whether they belong to HL learners.

## 3. Earlier research on FLP

Family language policy has been defined as “explicit and overt planning in relation to language use within the home among family members” (King et al., 2008, p. 907), integrating theory and data from the fields of language policy and child language acquisition (Fogle and King, 2013). The most cited model in FLP studies is Spolsky’s (2004, 2009, 2012, 2019) tripartite model, which comprises language ideology, language practice and language management. Language practice refers to how family members habitually interact with each other verbally, i.e., what choice they make from their linguistic repertoire. Language management is conceptualized as specific efforts or strategies parents use to modify or influence their language practice. Underlying these two components are language beliefs, also called language ideology, about operating language practice and language management efforts. This model sets a framework for research on parent–child interactions in immigrant families and child language development (Fogle and King, 2013).

Earlier FLP research highlighted parental perspectives, agency, decision-making and management of HL (King et al., 2008; Curdt-Christiansen, 2009; Kang, 2015). As the child caregiver, the parents usually make decisions and act as a model for the children in language use (Park and Sarkar, 2007; Chatzidaki and Maligkoudi, 2013; Zhu and Li, 2016; Shen, 2017). Parents’ language attitudes, cultural dispositions, language practices and strategies largely determine whether HL can be maintained in the younger generation (Park and Sarkar, 2007; Szecsi and Szilagyi, 2012; Shen and Jiang, 2021). The shift away from HL is more common in families with little-to-no overt planning by immigrant parents (Fogle and King, 2013).

These policies and practices, however, are neither static nor unidirectional. The critical role of children in shaping and reshaping parents’ FLPs has aroused scholarly interest (Fogle and King, 2013; Said and Zhu, 2019; Wilson, 2020; Smith-Christmas, 2022, etc.). The children could either negotiate, contest or resist the explicit policy decisions implemented by the parents, which in turn impacts their FLPs (Boyd et al., 2017; Revis, 2019). The parents have the good intention to socialize their children into HL usage; however, how the children feel, experience and react is of no less importance than what the parents are trying to implement (Wilson, 2020). Curdt-Christiansen (2009, 2014) and Curdt-Christiansen and La Morgia (2018), therefore, further develop Spolsky’s model of FLP by incorporating internal and external factors. Included internal factors are emotion, identity, family culture and tradition, parental impact belief and child agency (Curdt-Christiansen and Huang, 2020). Curdt-Christiansen and Huang (2020) defined child agency as “children’s active role in making decisions about patterns of family language use” (p. 178). They argue that child agency is noticeable but complex between the two generations and thus should be treated with careful consideration.

## 4. Chinese language education in Australia

The maintenance of Chinese as an HL overseas is complicated by the diversity of Chinese language varieties. “Chinese” is an ambiguous label when used to refer to language. The Chinese language consists of seven major “dialects” (Taylor and Taylor, 2014) or “language varieties” (Wiley et al., 2008), which are usually mutually unintelligible orally but share the same written form using Chinese characters (Li, 1994). Of the seven major “dialects,” Mandarin is the one with official status and the largest number of speakers in China. Apart from Mainland China, Mandarin is also officially used in Taiwan under the name of *Guoyu* (“national language”) and in Singapore under the name of *Huayu* (“Chinese language”) (Taylor and Taylor, 2014). Cantonese is referred to as a dialect within Mainland China, however, it is often referred to as “Chinese” language overseas. In this article, Mandarin and Chinese are used in an interchangeable manner referring to the official language used in China, Taiwan and Singapore.

In Australia, the number of Mandarin speakers has surpassed that of Cantonese speakers. The census statistics indicate that in 2011, the percentage of Australians speaking Mandarin at home is 1.6%, slightly higher than 1.2% of Australians who speak Cantonese. By 2016, among the Chinese Australians who make up 5.6% of the nation’s whole population, the number of Mandarin speakers (596,711) is more than twice the number of Cantonese speakers (280,943) (Australian Bureau of Statistics, 2017). Released in the most recent 2021 Australian Census, Mandarin continues to be the most spoken language other than English (685, 274), while Cantonese has been overtaken by Vietnamese (Australian Bureau of Statistics, 2022).

With the fast-growing Mandarin-speaking community, Mandarin has also been included nationwide as part of the Australian school curriculum, being placed among the top priority LOTEs (Australian Curriculum, Assessment and Reporting Authority, 2016). In the meantime, community language schools flourish in Australia, which greatly contributes to the maintenance of immigrants’ HLs and cultures. Each state is providing grants to these community language schools in support of their operations. By 2020, there have been nearly 100 Chinese (Mandarin) community language schools across Australia (Department of Education, Skills and Employment, 2021). This means that Australian-born Chinese have the opportunity to receive formal instructions in Mandarin *via* school language programs, community language schools or both. Considering the Chinese heritage background of this study, the researchers use weekend Chinese language schools when discussing community language schools in this article because they usually operate on both Saturdays and/or Sundays.

Despite these language opportunities both at the familial and institutional levels, language shift is still evident for second-generation Chinese immigrants according to previous studies, particularly in the area of the second generation’s literacy abilities (Clyne and Kipp, 1999, 2006; Chen, 2013; Hu et al., 2014; Shen and Jiang, 2021, etc.). Reading or writing in HL may resist language shift longer than merely a conversational level of HL for daily communication; however, full and literate proficiency in HL is difficult to achieve, particularly when HL differs so greatly from the socially dominant language. Adopting an ethnographic approach to three Chinese immigrant families, this study attempts to explore the FLPs upheld in these families, re-story the bilingual experiences of the Australian-born generation and provide implications for heritage language and cultural maintenance for a wider community. The specific research questions to be addressed are: (1) What FLPs were practiced by the parents in these three families? What are the differences among them? (2) How were the children responding and reacting to their parents’ FLPs? (3) What were their HL learning outcomes?

## 5. Research methodology

To gain an in-depth understanding of the abovementioned research questions, substantial fieldwork has been conducted in a Chinese community in Australia with qualitative data collection methods employed. The research methods are specified as follows:

### 5.1. Storytelling as a narrative inquiry method

Telling stories is a crucial qualitative approach to language research that provides a rich source of knowledge and meaning making (Dwyer and Emerald, 2017). Clandinin and Rosiek (2007) believe that the stories lived and told fill our world with meaning and help us build connections between each other in lives and communities. People’s daily lives are shaped by stories of who they and others are while they are recalling and interpreting their past in these stories (Connelly and Clandinin, 2006).

The telling of stories is a narrative reproduction of chronologically connected events of spoken or written texts relating to the significant lived experiences of the individuals who instill meaning in the world (Merriam and Tisdell, 2016; Nasheeda et al., 2019, etc.). Recognized as a unique type of narrative inquiry, storytelling emphasizes collaboration and engagement between researcher and participant to retell the participant’s past and present realities (Clandinin and Connelly, 2000). In narrative research, the process of crafting a story of the participant should involve a complex set of strategies and truthfully reflect the actions, choices and beliefs of the participant. It is also through this process that important clues about how individuals use their language(s) and engage in identity construction are revealed (Nasheeda et al., 2019).

### 5.2. Participants and ethnographic fieldwork

This study adopted an ethnographic approach to collect in-depth and multi-dimensional data from the participating families. It emphasizes an “emic or insider’s point of view” and endeavors to derive meanings and understandings of data through their engagement in the field setting (Mills et al., 2010, p. 596). In ethnographic studies, the researchers’ constant exposure to the community and sustained engagement with the participants are essential for understanding and interpreting what people actually do in their lives (Schensul et al., 2012). The ethnographic fieldwork for this study was conducted at a renowned weekend Chinese school in Brisbane, Australia. It is a non-profit community school specializing in teaching Chinese to children aged from 4 to 16 who are of various Chinese proficiency levels. Having been established over 15 years, the school is well known as the largest community-based weekend Chinese language school in Queensland. The fieldwork lasted for approximately 18 months, including 2 months’ unstructured observation at the research site as preparation and 2 months’ pilot study prior to data collection. During this course, the first author spent, on average, one day every weekend on the research site plus special days or festivals, where cultural events and performances were hosted by the weekend Chinese language school for all the learners and parents in its community. By doing this, the researchers aimed to gain a holistic view of what was happening on the research site and more insights into the participants’ experiences.

The three families involved in this article were epitomes of the 30 families the researchers recruited for a larger project. All the participants were given a Participant Information Sheet, a Chinese version for parents and an English version for children. The parents were asked to sign a Participant Consent Form for themselves and a Guardian Consent Form for their participating child before the commencement of formal research procedures. The protagonists of the three family stories reported in this article, i.e., Leo, Tracy and Anne (all pseudonyms), were studied as three typical cases out of the 30 child participants in that project because they represented the high, medium and low levels of HL proficiency outcomes, respectively, as evidenced in an oral and written Chinese proficiency test (Shen, 2017; Shen and Jiang, 2021; Shen and Jiang, 2022).

Data were collected through two formal interviews, i.e., one parental interview (approximately 1 h) and one child’s interview (approximately 40 min), family background information provided by the parents, and the notes taken by the researchers during the informal meetings with the participants. The formal interviews were semi-structured and targeted at eliciting in-depth information about the participants’ perspectives and practices in regard to their (children’s) language experiences. A list of 16 questions were pre-formulated as a guide for the interviews (see Appendices A, B), which involved a variety of sub-topics regarding FLPs, such as home language use, HL literacy practices, parents’ expectations, ethnic identity and exposure to the HL and its culture. These topics were elicited from various similar studies in the literature (e.g., Lao, 2004; Park and Sarkar, 2007; Curdt-Christiansen, 2009; Zhang and Slaughter-Defoe, 2009; Hu et al., 2014). The majority of the questions were open-ended and aimed to guide the participants to report on their past experiences, stories and perceptions of various aspects of Chinese language maintenance.

In addition, the researchers had at least two informal interviews with each family before the formal interview so that sufficient familiarity and trust had been fostered before the formal interview started. The first author received the invitation from all the three families to visit their home, which demonstrated a trustworthy relationship between the researchers and the participants. Only one parent from each family was involved in the interviews, and coincidentally, all three parents who volunteered were mothers. The profile of the child participants and their parents’ background information are presented in Tables 1, 2, respectively.

**Table 1:** Profile of the child participants.

	School year level	Age	Birth country	Gender	Siblings
Leo	5	11	Australia	Male	1
Tracy	5	11	Australia	Female	1
Anne	5	10	Australia	Female	0

**Table 2:** Profile of the parent participants.

	Birth country	Hometown	Length of living in Australia
Jessica (Leo’s mother)	China	Qingdao (north)	15 years
Fiona (Tracy’s mother)	China	Shanghai (southeast)	20 years
Anne (Chloe’s mother)	China	Guangzhou (south)	15 years

During interviews, all the parent participants selected Mandarin Chinese as their preferred language while all the child participants voluntarily chose English. Only the formal interviews were recorded and transcribed verbatim. The informal interviews were unstructured, giving participants more freedom and spontaneity to share their own stories and feelings. The summary of each informal interview and notes taken during the interviews were used as complementary data for constructing the protagonists’ stories.

### 5.3. Constructing stories from the data

**Figure 1:**
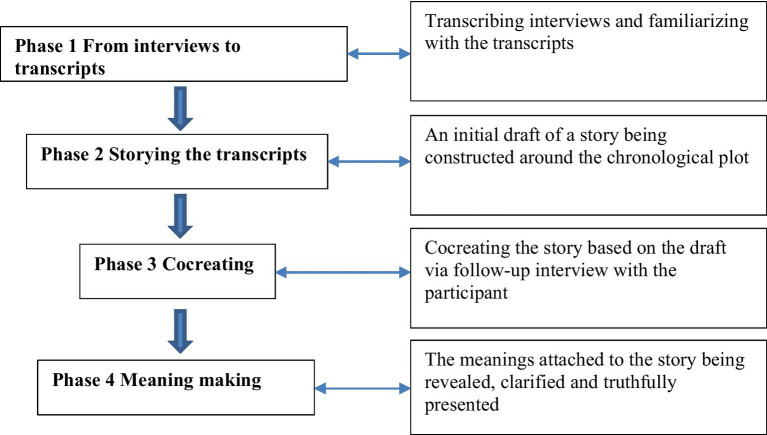
A four-phrase framework for constructing the stories.

Stories need such essential elements as characters, settings, actions and experiences of an individual, which need to be recognized, analyzed and retold in chronological order (Clandinin and Connelly, 2000; Clandinin and Rosiek, 2007; Nasheeda et al., 2019, etc.). The current study extracted these elements from the fieldwork data, emplotted them and turned them into a coherent family story for each participant. Emplotment is crucial for crafting stories from interview transcripts (Czarniawska, 2004), through which a sense-making mechanism needs to be established on how all these elements are threaded into an organized and meaningful narrative. During this process, the four-phase progression as a multimethod approach to narrative analysis put forward by Nasheeda et al. (2019) was adopted comprising: (1) from interviews to transcripts; (2) storying the transcripts; (3) cocreating between the researcher and the participant; (4) meaning making. These four phases are briefly illustrated in Figure 1. By applying this approach, the study attempted to create a holistic story of the lived experiences of the participants while extracting the segments or episodes from the data.

Families have a shared repertoire of stories around language experiences. Parents and children not only have their collective lived language experiences but also their own individual experiences to draw on in their storytelling (Obojska and Purkarthofer, 2018). The researchers of this study, therefore, interviewed both parents and children. The process of retelling each family story from the data is not only a reflection of the familial language ideologies and language practices but also the individual family member’s own experiences and feelings. To maintain objectivity and avoid researchers’ bias, the stories reconstructed were all brought back to the participants to confirm whether the emplotment accurately reflected the participants’ experiences and voice. The participants were requested to make corrections or additions where they disagreed.

## 6. Findings

The study reveals three distinct family stories with respect to the home language environment, parental language ideologies and practices as well as the children’s experiences, feelings, and reactions. The three stories were presented in this section.

### 6.1. Leo’s family story – “I am proud that I can speak Chinese”

Leo was a Year-5 student at a local primary school, where there was a high proportion of students whose parents migrated from China. Like many of his Chinese friends, he grew up speaking two languages, English at school and Mandarin Chinese in the home environment.

Leo’s parents were originally migrating from a northeastern coastal city in Mainland China. His father was a businessman travelling back and forth between China and Australia, while Leo’s mother Jessica stayed mostly in Australia with her two children. Normally once every year, Leo went on a short visit to China with his parents. He loved those trips that he described as being “cool” and “impressive.” He mentioned that he was deeply impressed by the delicious food, numerous tourist sites and a wide range of toys made in China. He felt he would never get bored during those trips because there were always exciting things in China that he had never seen or experienced in Australia. When Leo could not visit China, his most unforgettable moments were when his father returned from China with a great variety of gifts and fun stories.

Leo had a younger sister who was almost 3 years younger than him. He described his daily interaction with his sister this way,

When I talk to my sister about our school, games and cartoons, we all speak English only. It is troublesome and weird to translate them into Chinese and my Chinese is not good enough to say much about these things. And my sister’s Chinese is even worse than mine. She could only say “吃饭了(dinner time)”, “睡觉了(go to bed)”, “上学了(go to school)”, and nothing else, so we normally just talk to each other in English. (Leo)

Although Leo demonstrated the highest Chinese language proficiency level among all the 30 participants in the larger project (Shen and Jiang, 2021), he was still used to speaking English with his sister. It can be inferred from the statement above, the siblings did not have the sufficient knowledge of the Chinese language to carry out in-depth communication on sophisticated topics or things happening in their schools.

Leo’s grandmother often visited them and helped to take care of Leo and his younger sister during her visit. Jessica spoke highly of the grandmother’s role in Leo’s Chinese language development before he started school, not only in oral communication in Mandarin but also in his Chinese literacy. Jessica recalled that Leo spent a larger quantity of time with his grandma than his younger sister did. When the grandmother was around, she often taught him Chinese rhymes and poems while playing with him. Although she had a slight Qingdao accent, she spoke Mandarin in an easily intelligible way. After his grandfather passed away, Leo’s grandmother came to live with them in Australia permanently. Since she was advanced in years, she spent most of the time now at home, watching television or sitting in the backyard. Leo often sat together with her watching television, a habit developed since he was very young. He always respected her choice of the programs, such as the news, entertainment, drama or whatsoever it was that she loved watching in Chinese. He expressed compassion and care for her grandparents because in his eyes, his grandmother was lonely and had no friends to communicate with. Television was her best companion. Jessica believed that watching Chinese television programs with his grandmother was of great help to nurture Leo’s Chinese literacy. She was amazed by the new words or sentences that Leo occasionally picked up from these programs. However, she had also recognized that the older the children grow, the less they communicate with their grandmother.

Leo was sent to weekend Chinese language school when he was 5 years old. He could not remember whether he liked it or not at the beginning, but after years of going there every weekend, he had developed friendships with other kids there that motivated him to keep going. He had several best friends there who went to the same primary school as him. In the day school, they spoke English most of the time, but occasionally he and his best friends joked with each other and shared secrets in Mandarin. On these occasions, usually his non-Chinese-background classmates did not know what they were laughing at. He commented that it was funny to do so, and it would be a great pity if he were not able to speak Chinese with his Chinese friends. He enjoyed playing with them and would miss them on weekends if he stopped going to weekend Chinese language school. He remarked, “I am proud that I can speak Chinese.”

In addition to attending weekend Chinese language school, Leo started learning Chinese as a school LOTE subject in Year 4. Due to his Mandarin-speaking background and years of learning experiences at weekend Chinese language school, he deemed it a waste of time for him and his Chinese friends to sit in the LOTE class, but the school did not offer them the choice of another language or a Chinese class of a more advanced level. He was looking forward to high school, when he could choose a European language, such as Spanish, French or German, for LOTE.

Growing up in a mainly Mandarin-speaking home environment, Leo felt it was easy to understand and speak Mandarin. However, Leo also mentioned the frustration of reading Chinese books, which involved memorizing a huge number of Chinese characters that he did not know. He tried to read Chinese books annotated with *Pinyin*; however, not all the books had *Pinyin* on top of the characters. Leo considered it troublesome and time-consuming to look them up in a dictionary one by one. Sometimes he would be discouraged from reading a Chinese book by the unknown Chinese characters. His favorite Chinese stories include *Xi You Ji* (The Journey to the West) and *San Guo Yan Yi* (The Three Kingdoms). He commented that it was much more fun to read these books than to merely copy the Chinese characters as part of his homework. In addition, he also expressed his reluctance in writing Chinese because it required a great deal of time and effort to practice.

Jessica disclosed her satisfaction with all the progress her son had achieved in learning Chinese. She remarked, “it is a very wise choice to get her son immersed in a formal Chinese learning environment like weekend Chinese language school.” Although she prioritized oral communication ability in Mandarin, she had been convinced by Leo’s experiences that it would be better to have some knowledge of Chinese literacy than to have none at all. On their return trips to China, Leo could read at least a few public signs on the street and would not be lost. Furthermore, each time she returned from China, she purchased Chinese books for Leo. Instead of buying the sophisticated original Chinese novel, Jessica found a simplified children’s version, annotated with *Pinyin* and illustrated with pictures, which had successfully aroused Leo’s interest. Leo’s father often discussed with Leo an episode or a character in Leo’s beloved Chinese novels. All this extra support proves to be beneficial in motivating Leo to keep learning Chinese.

Jessica holds that the world has become a global village, where bilingual or even multilingual global citizens are in great demand. The descendants of the immigrants should become confident English-speaking global citizens and cherish their roots in their heritage language and culture simultaneously. With this earnest wish, she insisted on speaking Mandarin at home and provided ample opportunities for Leo to progress in Chinese literacy.

### 6.2. Tracy’s family story – “I am still fighting with my parents about not learning Chinese”

Tracy was a Year-5 student in a Catholic school. Her parents migrated from Shanghai, a south-eastern coastal city in China. Tracy was the second daughter in the family. Her father was a businessman, and her mother was a housewife. Tracy’s mother, Fiona, demonstrated a distinct awareness of the potential economic value of being able to speak Mandarin. Seeing growing interest in learning Chinese worldwide, Fiona considered it a great shame if the second-generation Chinese Australians did not take advantage of their Chinese background and master the Chinese language.

However, Tracy was sent to childcare at 2 years old, which meant an early immersion for her in an English language environment. In Fiona’s memory, Tracy could already speak a good amount of English by the age of 4, but she had never been fluent in speaking Mandarin. Fiona did not take it seriously until Tracy started primary school. Fiona felt shocked and deeply concerned when all of a sudden, she could not hear Tracy speak Mandarin anymore. She regretted missing the best opportunities to enforce the rule of communicating in Mandarin at home before Tracy started school. She reflected that although she always spoke to her children in Mandarin, she usually allowed them to respond in English or a mix of Mandarin and English, particularly when she was in a hurry to get a response from them.

Fiona took Tracy back to China three times to visit her grandparents and other relatives. The first visit was before Tracy went to childcare, so Fiona said Tracy did not have any memory of that experience. Fiona recalled their second visit to China, when Tracy could barely communicate with her grandparents or their relatives, which made Fiona and her husband realize the urgency of cultivating Tracy’s communication abilities in Mandarin. Fiona said, “You can communicate with anyone in China if you are able to speak Mandarin, but you can only communicate with local Shanghainese if you speak the Shanghainese dialect.” In addition, she was afraid that speaking the Shanghainese dialect might make her daughter more confused in learning to read and write the standard written language. Therefore, Fiona and her husband decided to consciously use more Mandarin in their daily conversations and deliberately forced Tracy to speak Mandarin. To their disappointment, however, Tracy had never been able to conquer the barrier of communication in Mandarin.

Fiona described a scenario of her two daughters watching Chinese cartoons, which left a deep impression on her. She recalled,

The sisters often discussed the plots and characters in English while they were watching the Chinese cartoons. It appeared strange and funny to me that their brains worked like translation machines in front of the television with Chinese input from one end and then English output from the other end. (Fiona)

This observation made Fiona greatly concerned. She realized that her daughters could only partially guess what was happening in the cartoons but were unable to express themselves in Mandarin.

In Tracy’s words, speaking English was definitely her first choice because she felt anything related to Chinese was hard. She had never been good at Chinese while she excelled in English. She said she spoke Mandarin only when she had to, for example, in Chinese classes or when her parents asked her to. She was keenly aware of her parents’ pretense when they said to her “Speak Chinese! I cannot understand you.” She mentioned she was already very used to the pattern of responding in English while her parents talked to her in Mandarin. When they suddenly showed this reaction saying that they could not understand her English, it struck Tracy that they were faking their desire and being ridiculous. Therefore, she either ignored them or gave a quick response to end the conversation.

Having realized Tracy’s remarkable shift to English, Fiona followed her friend’s advice to enroll Tracy in the weekend Chinese language school when Tracy reached five. She called this decision a milestone on Tracy’s struggling journey of learning Chinese. Tracy was unwilling to take on extra learning on weekends; however, Fiona used any incentives she could think of to keep Tracy going, such as candies, gifts and playdays with friends. After approximately 1 year, Fiona no longer heard any arguments or excuses from Tracy about not attending Chinese classes.

Tracy gradually became accustomed to going there because she could meet her friends every weekend. However, she still occasionally had the impulse to quit when feeling overwhelmed by Chinese characters. When her mother Fiona forced her to do Chinese homework, it always made Tracy feel depressed or miserable. She even described doing Chinese homework as a nightmare, which she tried to escape or postpone to the last minute. Tracy seemed not to appreciate her mother’s help with her Chinese homework. She confessed a feeling of being pushed, and her mother was not as well-tempered and patient as was her teacher. She wished she could have more fun reading and writing in Chinese, but in fact, it turned out to be frustrating and sometimes even hopeless. She found that when she taught herself French on the iPad, she enjoyed learning a few words every now and then. However, in learning Chinese, she only felt bored and upset. Although it was an unnegotiable requirement of her parents, Tracy was “still fighting” with her parents about learning Chinese.

Tracy started with Chinese LOTE classes in her primary school from Year 3, and this continued into Year 4 and Year 5. She found the Chinese classes at school to be quite easy and relaxing. Most of her classmates were “Aussies,” who was learning Chinese from scratch. She often became bored when she had to do the same Chinese exercises as the rest of the class. However, she enjoyed being an assistant to her Chinese teacher, correcting her classmates’ pronunciation and helping them write Chinese characters. She felt she was smarter than the rest of the class because she learned Chinese faster than them. She was once even a “temporary teacher” when her Chinese teacher was away on sick leave. An Australian teacher in her school helped her to organize the class while she showed her fellow classmates what to do. Tracy recognized that all these positive outcomes were attributed to her hard work at weekend Chinese language school.

Upon her experiences of raising her two daughters, Fiona concluded that the earlier a child starts weekend Chinese language school, the easier it is for the parents to persuade the child to follow their decision. The difficulties of doing so increase as the child gets older. It is better to get children used to taking Chinese classes when they are small, so they naturally accept it as part of their lives. Although Tracy is still struggling in learning Chinese, Fiona holds that “it is worth the efforts we are putting in” and shows pride in Tracy’s progress. Furthermore, Fiona found it extremely difficult to persuade her elder daughter to continue with weekend Chinese language school because she was involved in more extracurricular activities and had more academic pressure in high school. In Fiona’s opinion, it is ideal for the children to take an early start in learning Chinese and build a solid foundation in Chinese literacy during the primary school years.

### 6.3. Anne’s family story – “I will never be able to speak Chinese”

Born and raised in Australia, Anne was the only child in her family. She was studying in a Catholic school. Approximately 90 percent of the students in Anne’s school were from an English-speaking background. Anne was the only student of Chinese heritage in her class. She did not have any friends with a Chinese background, and her cousins, who could speak Mandarin, Cantonese and English, were all living in Sydney.

Anne’s parents originally came from Guangzhou in Mainland China, where the local spoken variety of Chinese used is Cantonese. Though having admitted to being a native Chinese speaker fluent in both Mandarin and Cantonese, Anne’s mother, Chloe, formed a habit of communicating with her daughter in English. Chloe argued that since Anne was born and raised in Australia, it was natural for her to use English more often, which had naturally become her first language. Chloe could not remember when this pattern of communication started, but in her memory, Anne never voluntarily spoke Mandarin or Cantonese. Before Anne went to childcare, Anne’s great grandmother helped to take care of Anne while Chloe was busy with work, so at that time Anne learned a few Cantonese words from her great grandmother. However, Chloe never meant to teach Anne Cantonese, so Anne gradually developed the pattern of only speaking English both outside and at home. In addition, Chloe had concerns over her own strong accent while speaking Mandarin, so she did not want her daughter to be influenced by her poor pronunciation of Mandarin. She tended to associate accents with the negative impression a person might leave on others. Chloe did not teach her daughter Cantonese purposefully because she did not attach any educational value to Cantonese. In her opinion, Cantonese was only used for informal communication purposes.

In Anne’s recollection, when she visited her grandparents and other relatives in Sydney, she usually had little oral communication with them because they hardly spoke any English. She only played with her cousins who mainly spoke English like her. At family gatherings, when their relatives spoke Cantonese or Mandarin, Anne needed her parents to translate the key messages of their conversation. Therefore, Anne said she normally shied away from these occasions because she felt embarrassed and bored when she could not understand what was happening in their conversations.

Anne recalled she started her first attempt at learning Chinese in Sydney at the age of six, which ended soon partly because she could not understand much Chinese and partly because they were leaving for Brisbane. Later, after Anne’s family settled down in Brisbane, Anne said her mother tried to persuade her to take Chinese classes again. At first, she cowered away from learning Chinese due to her initial unsuccessful experiences. Then, in the first term of Year 5, Anne was finally convinced by her mother to make another attempt. She agreed with her mother that it was beneficial to her future if she could know enough Chinese to communicate with more people and have more opportunities to get a well-paid job.

When Chloe urged Anne to make the second attempt at taking Chinese classes, she found Anne took the learning tasks more seriously and exerted more effort in her homework than previously. However, Anne still struggled in the learning process and could not achieve much progress in either communication or Chinese literacy. She revealed,

My mother asked me to give it a try. I agreed. I really tried hard to understand the teacher and to learn some Chinese, but it did not work for me. I often got distracted in class, because I did not know what the teacher was saying. I felt I did not know a single thing about the Chinese language. It was too boring and depressing for me to sit in the Chinese class, so I gave up. (Anne)

Anne felt it was “boring” and “depressing” to learn Chinese, because she can hardly understand what the teacher was saying. Her parents only spoke English with her, neither Mandarin nor Cantonese, which she knew they could speak. She heard her parents talk in Mandarin with their Chinese friends and relatives, but she said she could not understand a single thing they were saying. She expressed that she did not want to try learning Chinese again, because it always reminded her of the shameful experiences of knowing nothing in the Chinese class. Anne also reported, she was taught six Chinese characters each week in class, including their *Pinyin* and how to write the strokes of each character in a correct way. She felt *Pinyin* was similar to English letters and, therefore, was more easily recognizable. However, learning Chinese characters was an insurmountable barrier to her. From her perspective, some characters had meanings while others did not, and one Chinese character had to be combined with other characters to make a phrase, which was totally confusing to her. Anne confessed that she could hardly read or remember any of the Chinese characters she had learned or understand the ways the Chinese characters are combined to generate meaning.

Anne confided that her parents did encourage her to learn Chinese, but they did not really offer her much help when she struggled with the Chinese classes and homework. She believed that other learners in her Chinese class had no problem understanding the teacher because they probably got used to their parents’ speaking Mandarin at home or they might have lived in China for a while. Her situation was totally different from that of her fellow classmates at the weekend Chinese language school, so she found it hopeless trying to keep pace with them. With little understanding of Mandarin, she always felt at a loss regarding what she should do and, therefore, constantly got distracted in class.

Sometimes when her mother did try to help Anne out with her Chinese homework, Anne had no idea at all about what she should do. Anne felt she did not have a single Chinese word in her mind, so it was impossible to manage her work. At first, her mother wrote down every answer for her to copy, but gradually, they abandoned this practice because both she and her mother found these efforts fruitless. Anne did not have access to Chinese television at home or any Chinese books. She had never traveled to China. In Anne’s own words, she was born in Australia, lived in Australia and was definitely an Australian. Feeling deflated by the failure of her two trials, Anne felt she would never be able to speak Chinese. It would be a waste of time and money if she idled away her time in Chinese classes with little progress. Finally, both Chloe and Anne agreed that it would be of greater importance to spend the same amount of time in English literacy skills and to achieve better academic results in school.

## 7. Discussion

The stories presented in this study revealed three distinct levels of Chinese language maintenance. They shared some commonalities, such as the same country of birth, the same year at school and, most importantly, the same cultural heritage. However, they differed greatly in their perceptions about learning Chinese, school experiences and home environments, as well as noticeably disparate FLPs. Their stories have demonstrated how different FLPs could impact children’s HL maintenance.

### 7.1. Parent agency of FLP

Parents’ action and intervention are essential in producing desirable effects in intergenerational language maintenance (Chatzidaki and Maligkoudi, 2013; Kang, 2015; Shen and Jiang, 2021). Parents play an essential role in establishing FLPs that explicitly or implicitly enhance HL development (Curdt-Christiansen and La Morgia, 2018). In this study, parents’ agency in managing children’s language use in the family domain was revealed in all three cases. The highest level of parental agency was demonstrated in Leo’s family, where the parents’ strong belief in the value of the Chinese language, close ties to their homeland, sustained use of HL with Leo, devotion to cultivating HL literacy and high expectations of bilingualism and biliteracy constituted important aspects of their FLPs. Moreover, only Leo’s parents adopted a variety of parental language management strategies in HL, such as providing books in Chinese classic literature, reading and discussing the characters with the child, and watching television in Mandarin Chinese. Home environments and activities for HL literacy are the most important part of language management, which can shape a child’s bilingual or multilingual development (Curdt-Christiansen and La Morgia, 2018).

In Tracy’s family, a lower level of parental agency was observed. Though emphasizing the communication ability in Mandarin Chinese, Tracy’s mother neglected the significance of Chinese literacy. Cultivating HL literacy means fostering the crucial ability to decode and encode an HL text, in which values, beliefs, and cultural dispositions associated with the HL are usually embedded (Curdt-Christiansen, 2009; Shen and Jiang, 2021; Shen and Jiang, 2022). Home literacy practices in HL are explicit and overt efforts from parents to cling to their cultural roots and HL identity in addition to progress in the HL itself. Therefore, a lack of HL literacy practices is detrimental to HL development (Clyne and Kipp, 1999; Szecsi and Szilagyi, 2012; Kang, 2015; Curdt-Christiansen and La Morgia, 2018, etc.).

In addition, according to Tracy’s parent, HL was beneficial instead of being necessary; therefore, she lacked motivation, determination and persistence in making her child form the habit of speaking Mandarin at an early age. When she noticed Tracy’s slip into the habit of speaking English only, she started to regret not insisting on communication in their HL at home. At this point, she exercised her parental agency by asking for advice from her friends, enrolling Tracy in weekend Chinese language school and purposefully speaking more Mandarin with Tracy. However, her FLPs were not well planned, and not carefully implemented either.

Anne’s parent, Chloe, acted the least parent agency in HL maintenance in this study. Her HL practices and management efforts were irregular and irresolute. She treated learning Chinese as a trial rather than attaching personal, emotional or cultural values to it. No consistent and explicit FLPs in favor of HL have been observed, and home environments for HL, which include culturally related practices, literacy-related resources and parental involvement in HL learning (Curdt-Christiansen and La Morgia, 2018), are largely lacking in Anne’s case.

The disparities between the three stories have evidenced the remarkable contribution of family support to the child’s HL competence. Parents’ language ideology is one of the strong predicators of oral and literacy levels in HL (Kang, 2015). Family inculcation into the heritage culture, encouragement from parents in daily use of HL and familial HL learning are all significantly related to children’s successful language maintenance (Mu and Dooley, 2015). Furthermore, the quality of HL language input and the influence of HL literacy experiences demonstrate to be crucial (Sun, 2019). The early HL exposure, ongoing commitment to HL use and literacy-based HL activities initiated by parents are only noticeable in Leo’s story, which definitely contribute to his confidence and competence in HL. Reading and interactions based on reading not only strengthen the children’s HL competence and facilitate their language production, but also enhance their social–emotional and behavioral skills (Sun, 2019). This study indicates that a high level of parent agency and support in HL, particularly in HL literacy input, is highly beneficial to language maintenance (Sun, 2019; Shen and Jiang, 2021; Shen and Jiang, 2022; Sun et al., 2022).

### 7.2. Child agency in heritage language maintenance

Children’s language ideologies are shaped and negotiated in their everyday language practices at home with their parents. Immigrant parents tend to have the intention to transmit their HL and use explicit language practice and management strategies to influence their children’s language development (Park and Sarkar, 2007; Szecsi and Szilagyi, 2012, etc.). However, children may contest or resist their parents’ efforts and undermine their parents’ FLP (Mu and Dooley, 2015 Smith-Christmas, 2022), which was exemplified by Tracy’s and Anne’s cases in this study. Both Tracy and Anne demonstrated resistance strategies toward HL, such as using their preferred language, English, in response to their parents, trying to escape from Chinese homework or even quitting weekend Chinese classes, which was a clear indication of language shift. Tracy was keenly aware of her parents’ tricks when they said, “I cannot understand you,” and her reaction of ignoring or putting the conversation to an end was plain resistance to the use of HL. In other words, she was asserting her agency in choosing the linguistic norms that she preferred. Little agency of keeping HL was found in Anne’s case. It has been noted that the level of child agency coincidentally corresponds with the level of their parent agency. Where parents strongly initiate the agency of HL maintenance, more agency is observed in their children to continue the use and learning of HL. Initially, the children might just mimic their parents’ linguistic codes at a very early age, and when they get a little older, they are unwillingly forced to take HL classes. However, over time, agency emerges and develops when children start to take the initiative in HL use and learning.

Compared with Tracy and Anne, Leo played an active and cooperative role in HL socialization and language maintenance at the familial level. Children’s agentive use of HL significantly contributes to the successful implementation of FLPs (Smith-Christmas, 2022). Leo’s agency was not only constructed and revealed in the reported daily interactions with his family members in HL but also in literacy practices, such as taking Chinese classes, reading Chinese stories and writing Chinese homework. Ideally, children are not passive followers but active contributors or collaborators of their parents’ FLPs, who have the ability to make sense of what they are doing, contribute to language socialization and formulate metalinguistic comments in learning and using HL (Revis, 2019). As previous researchers argue, children can “exert their agency to make creative use of heritage language and the mainstream language” (Curdt-Christiansen and Huang, 2020, p. 182). Leo’s story contained an interesting episode of creative use of HL among peers. Leo and his Chinese friends at school, though speaking English dominantly, could occasionally entertain themselves by joking with each other and sharing secrets in their HL. The same cultural background and the common experience of attending weekend Chinese language school must have enabled them to assert “in-groupness” and form intimate bonds between them through a way of communication unique to this group of bilingual children. They can “mobilize their multiple (and developing) linguistic repertoires creatively to assert their agency in language use and socialization” while others cannot (Said and Zhu, 2019, p. 773). This episode appears to be a casual and inconspicuous occasion of child HL use; however, it may trigger quality changes in the process of child HL development because this creative use of HL with peers in the mainstream language environment is child-initiated. This indicates that child autonomy in language decision-making starts to emerge in their socialization.

Many researchers regard language acquisition and language socialization as an integrated process (Fogle and King, 2013; Said and Zhu, 2019; Smith-Christmas, 2020). That means the acquisition of HL is not merely associated with formal language learning in classroom settings, focusing on various linguistic components and language skills, but more importantly, happens informally and unknowingly with different family members at home and various social partners in the communities (He, 2008). In this study, the learners’ socialization with peers, e.g., siblings and schoolmates, were showcased in Leo’s and Tracy’s stories. The episode of “joking” and “sharing secrets” in HL between friends at school reported by Leo and Tracy’s experience of being a “temporary teacher” to her “Aussie” classmates both evidenced child agency in HL use and socialization. The impact of these experiences on learners’ path of bilingual development is long-lasting and truly beneficial. However, this study also found a minimal level of HL use in the daily interactions between siblings. This could be attributed to the fact that English is the main language of the education they receive, so they absorb in all new knowledge through English.

Child agency in HL literacy was discerned only in Leo’s case. He disclosed his struggle with the daunting task of learning Chinese characters and frustrating reading experiences without *Pinyin*. Despite this, he still loved the Chinese novels that appealed to him and discussed an episode or a character from these novels with his father. Agency was seen to be deployed in coping with all the difficulties that confronted Leo and be strengthened day in and day out to successfully manage HL use, either in an oral or written context.

### 7.3. Harmonious development in HL

Heritage language maintenance, as argued by many researchers, contributes to a harmonious and intimate relationship in immigrant families (Tannenbaum and Howie, 2002; Curdt-Christiansen and Huang, 2020). In contrast, maintaining harmony in implementing FLPs is also of importance to child language maintenance. Conflicts of identity and cultural values between different generations are inevitable since their encounters and experiences vary greatly (Curdt-Christiansen and Huang, 2020). How can these conflicts be melted away by harmonious FLPs in immigrant families?

As shown in Tracy’s family, they have, for years, formed a pattern of the parents speaking Mandarin and the child responding in English. When the parent alarmingly realized that her daughter was likely losing the HL, they tried to break this pattern by pretending to have not understood and making their daughter repeat in Mandarin. Harmony between the parents and the child was disrupted when the child saw through their disguise and was unwilling to continue the conversation. Another thing that affected the harmonious family relationship was the impatience and bad temper Tracy’s mother showed when Tracy suffered from doing her Chinese homework. There was a lack of in-depth parent–child communication about which part was too difficult for Tracy to complete on her own, what kind of support she specifically needed to overcome the difficulties and what might be easy and fun to do to balance out Tracy’s frustration in doing her Chinese homework. To rebuild the harmony, parents may need to adjust their language maintenance strategies, which are more likely to arouse their child’s interest in learning HL.

Heritage language is not just a connection between parents and the child but also serves as a bond with the grandparent generation or the extended family (Zhu and Li, 2016). In this study, Anne’s parents selected English—Anne’s preferred language—for daily communication and respected Anne’s choice of giving up on Chinese classes, which seemed to have created a harmonious monolingual environment. However, there were two points in the story that might become causes of future disharmony. First, Anne recounted her feelings and experiences in weekend Chinese classes, including what struggles she had undergone and why she suffered much more than other learners in class. Though still a child, she was keenly aware of the little HL support she gained from an English-speaking home environment. She was even observant and analytical of her problems with Chinese learning. She realized it was her parents who needed to take the blame for not teaching her anything in Chinese. Second, Anne had hardly any communication with her extended family in Sydney because most of the time they spoke Cantonese or Mandarin for family gatherings. She heavily relied on her parents’ translation or simply shied away from their conversations, feeling bored and embarrassed. Without HL, there was no way for Anne to establish affectional bonds with her extended family.

In contrast, the harmonious relationship between Leo and his family members, including his grandmother, can be summarized in the following two cues: first, growing up in a Mandarin-speaking home environment, he had already been accustomed to using the HL with his family members. No complaints were heard during several meetings with Leo about the inconvenience or difficulties of speaking Mandarin in daily life. The harmonious relationship gradually formed in a natural way they communicated in HL and in the discussions between the parent and the child on their beloved characters or fun episodes in stories. Second, Leo’s connection with his grandmother was also an indispensable part of the harmonious family relationship. Although Leo had less communication with his grandmother as he grew up, his understanding of her physical conditions, sympathy for her loneliness, and the actions of accompanying and caring for her remained a natural habit formed when he was small. The connection between the younger generation and the grandparent generation in the immigrant family relies heavily on HL, which serves as an expression of love and a bond of affection (Zhu and Li, 2016).

### 7.4. Family language policy—Mandarin over another “dialect” or “language variety”

Each of the parents in this study could speak a dialect—indigenous Chinese language variety spoken in their hometown in China, i.e., Qingdao dialect, Shanghainese dialect and Cantonese (in Guangdong Province), respectively. However, their preference for Mandarin as an HL over their own dialect for the second generation to maintain is evident in the stories. Leo’s and Tracy’s parents prioritized Mandarin in their communication with children at home. Although Anne’s parents chose English as the language for communication with their daughter, they still wished that Anne could understand Mandarin and achieve some Chinese literacy by taking weekend Chinese classes. Leo and Tracy were sent to weekend Chinese language schools at a relatively early age and spent years learning Mandarin and Chinese literacy.

This may be unique to Chinese language maintenance. People from different parts of Mainland China speak mutually unintelligible “dialects,” which are regarded as different language varieties; however, Chinese people are reluctant to call them different languages (Taylor and Taylor, 2014). With the diversity of dialects, there is only one written language in China, which uses Chinese characters as its writing system (Wiley et al., 2008). Mandarin is the corresponding spoken form of this written standard; therefore, parents attach political and educational value to Mandarin. As the official language variety of the Chinese government and the medium of instruction in schools, the parents were keenly aware of the potential advantages that Mandarin could bring to their children. This was confirmed by all the parents in this study who claimed themselves to be native speakers of Mandarin, though they all had their own dialects. They all wanted their children to inherit Mandarin and a certain level of Chinese literacy.

In Australia, Mandarin has also gained its place in the Australian language curriculum, which recognizes that learners bring their own linguistic and cultural background to their education (Australian Curriculum, Assessment and Reporting Authority, 2016). Together with community Chinese language schools, Mandarin as a LOTE would inevitably serve as an invisible force that pushes parents’ preference toward Mandarin. In Leo’s and Tracy’s cases, they not only received formal instruction of Mandarin in weekend Chinese language school but also in the school LOTE program, which would certainly be conducive to their maintenance of HL and culture.

## 8. Conclusion

By re-storying the three participants’ different life trajectories and language experiences, this study has presented a rich, multi-faceted and nuanced picture of FLPs in different family contexts. The stories highlighted the significance of agency in FLPs. This study suggests that more attention should be directed to the agency of children in FLPs. Child’s active cooperation, agentive use of HL in the home domain and creative use of HL in socialization with peers are strong indicators of successful FLPs in Leo’s language maintenance story. However, child agency does not come from nowhere. While parents initiate agency in formulating and implementing FLPs, children continue exercising the agency with their own understanding and creativity. In addition, a harmonious relationship, either in the nuclear family or in the extended family, incubates HL development in the younger generation. The Chinese language programs in community language schools and primary schools play a fundamental role in cultivating Chinese literacy, which will sustain FLPs and language maintenance in the long run.

The current study draws on a small sample of three distinct family contexts. The researchers expect to investigate a wider variety of familial contexts in the future and delve deeper into the agentive and creative use of HL by the younger generation in social and emotional interactions. Moreover, storytelling, as a unique research method of narrative inquiry, could be used more widely in HL studies. Long-term collaboration and engagement between the researcher(s) and the participants would be ideal to unpack the multilayered and complex process of HL development in children.

## Data availability statement

The raw data supporting the conclusions of this article will be made available by the authors, without undue reservation.

## Ethics statement

The studies involving human participants were reviewed and approved by the University of Queensland. Written informed consent to participate in this study was provided by the participants’ legal guardian/next of kin.

## Author contributions

CS and WJ: study conception, design, analysis, and interpretation of results. CS: data collection and draft manuscript preparation. All authors reviewed the results and approved the final version of the manuscript.

## Funding

This article preparation was supported by the Youth Foundation of Humanities and Social Sciences of the Ministry of Education in China (grant number 21YJC740043).

## Conflict of interest

The authors declare that the research was conducted in the absence of any commercial or financial relationships that could be construed as a potential conflict of interest.

## Publisher’s note

All claims expressed in this article are solely those of the authors and do not necessarily represent those of their affiliated organizations, or those of the publisher, the editors and the reviewers. Any product that may be evaluated in this article, or claim that may be made by its manufacturer, is not guaranteed or endorsed by the publisher.

## Footnotes

^1^LOTE was employed in the past Australian Censuses as an umbrella term for all languages other than English spoken in Australian households. The Australian Curriculum for Languages nominates LOTE as a compulsory language subject that requires a minimum of 350 h of study in primary years (from Foundation to Year 6) and 350 h in Years 7–10.
