# Supplementary material for: Parents’ planning, children’s agency and heritage language education: Re-storying the language experiences of three Chinese immigrant families in Australia
Source: Front Psychol. 2023 Jan 6;13:1083813. doi: 10.3389/fpsyg.2022.1083813 (PMC9881589; doi:10.3389/fpsyg.2022.1083813)
Supplement: Supplementary file 1 [file Data_Sheet_1.docx]

**Appendices**

**Appendix A: A Question Guide for the Semi-structured Interview with Parents**

Q1. Do you want your child to speak both English and Chinese? Why?

Q2. Do you think it is important for your child to learn Chinese? Why?

Q3. Do you think it is important for your child to be able to read and write in Chinese? Why?

Q4. How will you rank the importance of the four skills, i.e. listening, speaking, reading and writing when your child learn Chinese? Why?

Q5. Do you think it is necessary to send your child to Chinese classes in school or Weekend Chinese Language School? Why?

Q6. Would you describe yourself mainly as

A. Chinese B. Mainly Chinese C. Half and half

D. Mainly Australian E. Australian

Q7. Do you think a person has to be able to speak Chinese to be a real Chinese?

Q8. What is your expectation of your child’s Chinese proficiency?

Q9. Do you insist on speaking Chinese with your child? Besides you, who else speak Chinese with your child?

Q10. What resources or opportunities have you provided to your children for learning Chinese (e.g. buy audio/visual materials like books and cards, join in Chinese community activities, get together with Chinese friends, make use of media and technology, etc.)

Q11. What have you done to help your child learn Chinese in any or all of the following aspects: listening, speaking, reading and writing?

Q12. What do you think is the most difficult in maintaining Chinese in your child?

Q13. When your child doesn’t speak Chinese at home, what will you do?

Q14. Is your child attending or has he/she attended any class learning Chinese? For how long? Are you planning to continue enrolling your child in this kind of Chinese class in the next few years?

Q15. What have you done or what decision have you made that has the greatest influence on your child’s Chinese learning?

Q16. What else do you want to add?

**Appendix B: A Question Guide for the Semi-structured Interview with Children**

Q1. Do you want to speak both English and Chinese? Why?

Q2. Do you like learning Chinese? Why?

Q3. Do you like reading and writing in Chinese? Why?

Q4. Would you please put in order the four language skills in Chinese learning, i.e. listening, speaking, reading and writing, according to your preference? What are the reasons for your preference?

Q5. Are you attending or have you attended any Chinese class? For how long? Why do you take Chinese classes?

Q6. Do you like the Chinese classes? Why? Do you want to continue in the next few years? Why?

Q7. Would you describe yourself mainly as

A. Chinese B. Mainly Chinese C. Half and half

D. Mainly Australian E. Australian

Q8. Do you have any Chinese friends? What language do you usually use when you are playing together?

Q9. How well do you expect to learn or use Chinese?

Q10. Do your parents insist on speaking Chinese with you at home? Besides parents, who else speak Chinese with you (including family members here and relatives in China)?

Q11. Do you speak Chinese or English at home? When you don’t speak Chinese, what do your parents do?

Q12. What resources are available for you to learn Chinese? (e.g. audio/visual materials like books and cards, Chinese community activities, Chinese friends, media and technology, etc.)

Q13. What has either of your parents done to help you learn Chinese in any or all of the following aspects: listening, speaking, reading and writing?

Q14. Is it difficult to learn Chinese? What makes you think it’s (not) difficult?

Q15. What can you think of that made you feel like learning Chinese?

Q16. What else do you want to add?
